# The Use of *ex Vivo* Rodent Platforms in Neuroscience Translational Research With Attention to the 3Rs Philosophy

**DOI:** 10.3389/fvets.2018.00164

**Published:** 2018-07-19

**Authors:** Laura Lossi, Adalberto Merighi

**Affiliations:** Laboratory of Neurobiology, Department of Veterinary Sciences, University of Turin, Turin, Italy

**Keywords:** brain slices, organotypic cultures, neuronal circuitry, rat, mouse, cell transfection, 3Rs

## Abstract

The principles of the 3Rs—*Replacement, Reduction, and Refinement*—are at the basis of most advanced national and supranational (EU) regulations on animal experimentation and welfare. In the perspective to reduce and refine the use of these animals in translational research, we here discuss the use of rodent acute and organotypically cultured central nervous system slices. We describe novel applications of these *ex vivo* platforms in medium-throughput screening of neuroactive molecules of potential pharmacological interest, with particular attention to more recent developments that permit to fully exploit the potential of direct genetic engineering of organotypic cultures using transfection techniques. We then describe the perspectives for expanding the use *ex vivo* platforms in neuroscience studies under the 3Rs philosophy using the following approaches: (1) Use of co-cultures of two brain regions physiologically connected to each other (source-target) to analyze axon regeneration and reconstruction of circuitries; (2) Microinjection or co-cultures of primary cells and/or cell lines releasing one or more neuroactive molecules to screen their physiological and/or pharmacological effects onto neuronal survival and slice circuitry. Microinjected or co-cultured cells are ideally made fluorescent after transfection with a plasmid construct encoding green or red fluorescent protein under the control of a general promoter such as hCMV; (3) Use of “sniffer” cells sensing the release of biologically active molecules from organotypic cultures by means of fluorescent probes. These cells can be prepared with activatable green fluorescent protein, a unique chromophore that remains in a “dark” state because its maturation is inhibited, and can be made fluorescent (de-quenched) if specific cellular enzymes, such as proteases or kinases, are activated.

## Introduction

The principles of the 3Rs - *Replacement, Reduction* and *Refinement*—were first formulated in 1959 by Russell and Burch (http://altweb.jhsph.edu/pubs/books/humane_exp/het-toc). In the following decades the interpretation of the 3Rs has somewhat broadened (1) and the 3Rs have been redefined e.g., by the UK National Centre for the Replacement Refinement & Reduction of Animals in Research (https://www.nc3rs.org.uk/the-3rs). Nowadays Replacement falls into two categories: *full replacement*, where animals are fully substituted by human volunteers or established cell lines, methods *in silico*, or mathematical modeling, and *partial replacement*, where animals in lower positions along the evolutionary scale such as invertebrates or nematodes, or cultures of primary cells and tissues explanted from animals that are killed to purpose, and thus have not been employed in a scientific procedure that produces pain, are employed. When Replacement involves the use of lower vertebrates (such as fish) or invertebrates it must be stressed out that there is still an ongoing debate as regarding the capability of these animals to experience pain (https://f1000research.com/slides/5-1348). Reduction occurs when comparable levels of information from fewer animals are obtained. This often requires an improved experimental design combined with sound statistics. If avoiding the use of animals is impossible, Refinement refers to amelioration of scientific procedures and husbandry, so that animal welfare is improved and pain or distress reduced to a minimum.

A logical approach to the 3Rs in neurosciences requires that several issues specific to this discipline are carefully considered (2). It is today difficult to envisage the possibility to fully replace animals in the study of neuronal development, circuits, cognitive activities, degeneration, and repair. Although more feasible, at least on theoretical grounds, reduction also poses some practical problems to researchers. The widespread use of genetically-modified animals has permitted to develop very precious models to study the normal and pathological brain, but, at the same time, has dramatically increased the number of animals, mainly mice and fish, employed in experimental procedures. The augmented production and use of these new animal strains with e.g., gain/loss of function mutations or gene knock-out/knock-in has been, in fact, the main causal factor explaining the increased use of rodents (mice) in biomedical experiments, whereas the use of wild type animals has, on the other hand, been dropping steadily in the last decades (http://www.understandinganimalresearch.org.uk/animals/numbers-animals/#Trends%20over%20time). Refinement is beyond any doubt easier to be implemented. Amelioration in environmental conditions (enrichment), veterinary care and experimental procedures may quite easily reduce suffering and, hence, the ethical cost of research. At the same time, refinement produces scientific data of higher quality (3).

The use of animals in neuroscience research raises particularly harsh ethical issues. Many disorders of the nervous system are extremely painful and debilitative. The incidence of the major dementias, such as Alzheimer's disease, Parkinson's disease, motor neuron disorders, and chronic pain syndromes is increasing in the world population, but still adequate therapies are lacking. This, from one side, prompts the scientific community to intensify research, but, from the opposite, finds very limited understanding in the general public that is anxious to very rapidly benefit from the results of research. Neuroscience studies often require the imposition of very distressing and/or painful conditions to animals, or exerting shrewd or devious influence on their experience. This has a very high ethical cost that often appears to conflict with the utilitarian doctrine. The latter, on the fact that animals may experience physical and emotional pain, requires that any injury caused to animals is reduced to a minimum and counterbalanced by the advantages that can be obtained in return (1). At the same time, public acceptance (4) is contingent on the understanding that in order to achieve the greatest advantage for people, other living organisms, and the environment, the least possible quantity of pain will be provoked to the least possible number of animals (see https://www.ipsos.com/en/public-attitudes-animal-research-2016).

## *ex vivo* rodent platforms in neuroscience research

The primary goal of translational biomedical research is the quick and systematic transfer of the results of basic research to the human and animal clinics. To accomplish this goal it is required that basic research is organized in an integrated interdisciplinary manner in foreseeing potential clinical applications. The level of complexity of the animal body, from molecules to cells, tissues, and organs, requires the use of appropriate experimental tools enabling researchers to find scientifically sound responses to questions at the basis of their studies. Studies on individual cells (primary neurons or neuronal cell lines) *in vitro* have laid foundations to molecular and cellular neuroscience. However, when one comes to consider some of the aforementioned major issues in neuroscience research it becomes immediately clear that more complex experimental settings are required. Thus, animal models come into play and choice of the correct model(s) is of paramount importance for the success of translational studies. Although not always feasible, if one wants to avoid the use of intact animals, *ex vivo* platforms represent a good compromise under several aspects such as the reduction of the high costs of research *in vivo*, the possibility to perform medium-throughput screening of drugs and molecules of potential therapeutic application and, last but not least, the compliance with ethical issues.

The term *ex vivo* indicates a condition in which a fragment of a tissue or organ is explanted from the body and maintained in culture. In principle, this condition is very similar to that *in vitro*, but preserves, at least in part, the spatial, and cellular relationship between the different cellular types contained in the explant. Today, most *ex vivo* platforms take the form of a slice cut from the brain or the spinal cord and are valuable implements to study the circuits made by neurons in terms of the structure, physiology and pharmacology, as well as their response to experimental damage (5, 6). Slices can be fabricated with ease, retain tissue cytoarchitecture, offer the possibility to carry out several types of experiments very simply and permit an accurate regulation of the environment where cells are cultivated. Therefore slice-based platforms facilitate research aiming to investigate the correlations between structure and function, as well as the plasticity of neuronal interactions under normal and pathological conditions. Finally, these *ex vivo* platforms may be used for genetic and molecular screening in basic and translational research.

In neurobiology experiments, one usually employs two types of slice platforms (6). Short-living (a few hours) slices came into use mainly as substitutes of electrophysiological experiments *in vivo*, when the patch clamp technology was developed. Slices are ideal for studying the functional and pharmacological features of synapses, as terminals remain intact for at least 6–8 h in this type of preparations. Slices display greater mechanical stability over the intact animal and permit a careful control over the composition of the extracellular environment, these two features being at the basis of their widespread use in the last decades. In more recent times, *in vivo* whole-cell patch-clamp recording was substantially ameliorated and allowed investigating electrical behavior of single neurons as well the manner in which they operate in networks in the natural context of fully connected biological circuits (7). However, whole-cell patch-clamp recording *in vivo* is highly demanding in terms of equipment and technical skill, this explaining why acute slices still stand the challenge of time. To successfully deal with the main limit of acute slices, i.e., their short survival, organotypic cultures (OCs) have been developed as an alternative for long-term studies. OCs were produced starting from previous work done on cultured explants from various tissues or organs. Initial studies employed the so called roller tube technique, to permit access, and diffusion of oxygen in the thickness of tissue (5). Brain OCs, as routinely used today, were originally developed by Gahwiler (8) and retain, at least in part, several fundamental architectural characteristics of the tissue of origin, i.e., the connections among neurons, the quantitative ratio of different neuronal populations, and the relationship between neurons and glia (8). The subsequent instauration of a culturing technique based on the use of semiporous membranes (9), currently referred to as the semipermeable membrane technique or membrane interface technique, has further increased the use of OCs. According to the membrane interface protocol, the brain or spinal cord is sliced and a few slices are bedded onto a permeable membrane mounted on a plastic insert. The insert is then put in a Petri dish that is filled with the culture medium, so that slices receive nutrients from the underside and oxygen from the top. Thus they remain alive at the boundary between the O_2_/CO_2_ atmosphere of the incubator and the medium, within a thin fluidic sheet. The main methodological aspects related to the use of the semipermeable membrane technique have been recently reviewed (5).

In the fulfillment of the Replacement principle, OCs can be prepared from simpler organisms than mammals such as Drosophila (10) or tissues obtained from human patients (11, 12). However, it is immediately obvious that interest in their translational use is primarily related to Reduction and Refinement. Theoretically, OCs can be established from any species, but the vast majority of studies with OCs are carried out in rodents, which are the prevalent mammalian species employed in neuroscience research. This is particularly true for mice as the development of transgenic models has significantly increased their use (see above). Besides to small size and limited requirement of space, there are several advantages connected to the use of rodents in neuroscience research, but one has to consider carefully that some anatomical features are unique to each species (13), thus translation to humans is not always straight. The length of rodents' lifespan is important: rodents typically live less than two years, which, from one side, facilitates aging studies, but still represents a quite long temporal interval for follow-up. Therefore, alternative models in aging research have been proposed such as senescence-accelerated fish (14) or mouse (15) strains.

Rodent OCs can be prepared from virtually all areas of the brain (e.g., the olfactory bulb, cerebral and cerebellar cortices, striatum, hippocampus, basal forebrain, thalamus and hypothalamus, mesencephalon), the spinal cord, and the retina [see Table 1 in Lossi et al. (6) for references].

### Technical aspects

This article has not the purpose to address technical details for the preparation of acute slices or OCs. However, in considering the possibility to use these preparations according to the Reduction and Refinement principles it is convenient to recall here that acute slices and OCs display very different properties.

Slices have the advantage of behaving similarly to the *in vivo* situation. Molecules that are released as a consequence of the preparative procedure and may be toxic to the cells are usually washed out before electrophysiology or release experiments are carried out. For example, acute spinal cord slices can be subjected to challenge with capsaicin, the vanilloid extracted from the red hot chili pepper, to induce release of sensory neurotransmitters and mimic the central effects of inflammation (16). Thence, neurotransmitters are released in the artificial cerebro-spinal fluid (ACSF) where slices are maintained for short-term survival and can be quantitated e.g., with ELISA to study the mechanism and/or modulation of release (17).

In slices, cell survival is not relevant as well as the disconnection of terminals from their parent neurons. Thus it is commonly held that neuronal circuits are maintained unaltered, as well as the neurochemical content and subcellular site(s) of transmitter storage, in processes/terminals. However, some authors have reported that the temperature of the ACSF may influence dendritic spine plasticity (18) and synaptogenesis (6).

Slice thickness is also irrelevant to the success of experiments, unless it interferes with imaging of specific cell type(s) tagged with fluorescent reporter proteins (FRPs), calcium (19, 20)/potassium (21) indicators, or optogenetic probes (22).

Differently from acute slices, OCs require a period of adjustment to the *ex vivo* condition before use. After tissue explant and slicing, damaged cells release calcium, and glutamate at toxic concentration. As a consequence, there is a massive apoptotic cell death in the first days *in vitro* [DIV—see Figure 3 in Lossi et al. (6)]. Neuronal death can be, at least in part, counteracted/reduced by adding trophic factors to the medium. For example, addition of nerve growth factor (NGF) to basal forebrain OCs protects cholinergic neurons from death that follows axotomy during the slicing procedure (23). In parallel, in the same temporal interval, astrocytes proliferate to generate a more or less thick layer on the upper surface of the OCs [see Figure 2G in Lossi et al. (6)]. These examples illustrate why it is necessary to allow a stabilization period of 4-14 DIV before proceeding to experiments.

Another important issue is the age of donor (5). Explants may be prepared from embryos, postnatal pups (less than P12 in rats or mice) or adults. Embryonic, and postnatal tissues are easier to be cultivated, but there are increasing reports on the use of OCs prepared from adult or aged subjects (24).

#### Structural changes following disconnection of the explants from afferent/efferent fibers

Orientation of cut is very important in determining the subsequent anatomical and physiological properties of the slices, because, at least in certain instances, one or more specific pathways can be selectively spared and thus maintained in long-term OCs.

##### Axotomy of afferent fibers

**Figure 1:**
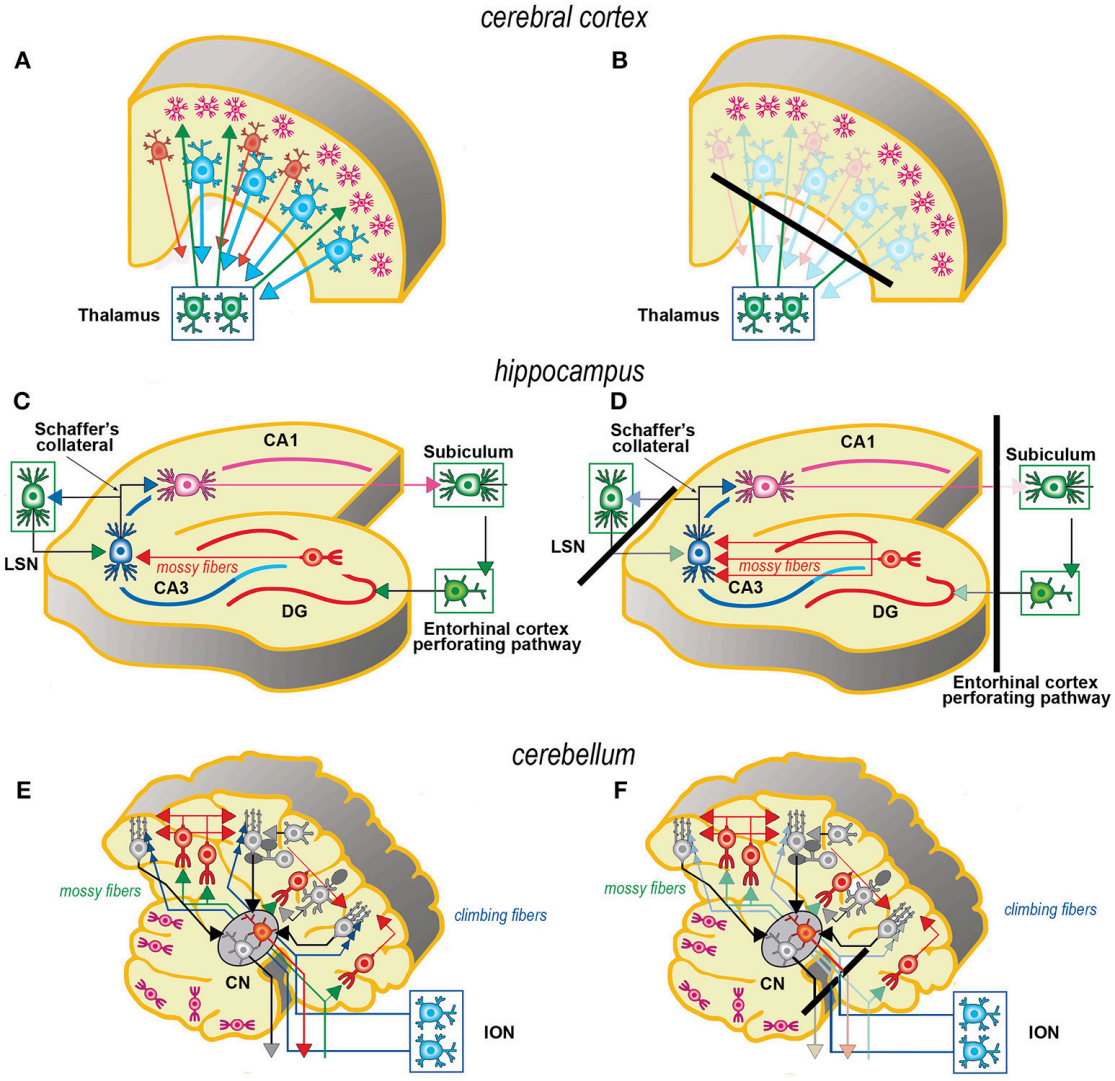
Simplified schematic representations of the effects of the slicing procedure on the preparation of acute slices (left) and OCs (right) from cerebral cortex **(A, B)**, hippocampus **(C, D)**, and cerebellum **(E,F)**. Only the main components of circuitry are depicted as well as the principal afferent and efferent connections. The lines of section (solid black bars) of the main fiber systems are represented only in the right panels to show the effects of resection onto the afferent and efferent systems (shadowed in comparison to the corresponding drawings at left). The magenta neurons in **E, F** represent the external granular layer of the cerebellar cortex, a temporary subpial cell layer that disappears in the mature cerebellum. CN, cerebellar nucleus; CA1, CA3, hippocampal subfields CA1 and CA3 (from Cornu Ammonis); DG, dentate gyrus; ION, inferior olivary nucleus; LSN, lateral septal nucleus.

**Figure 2:**
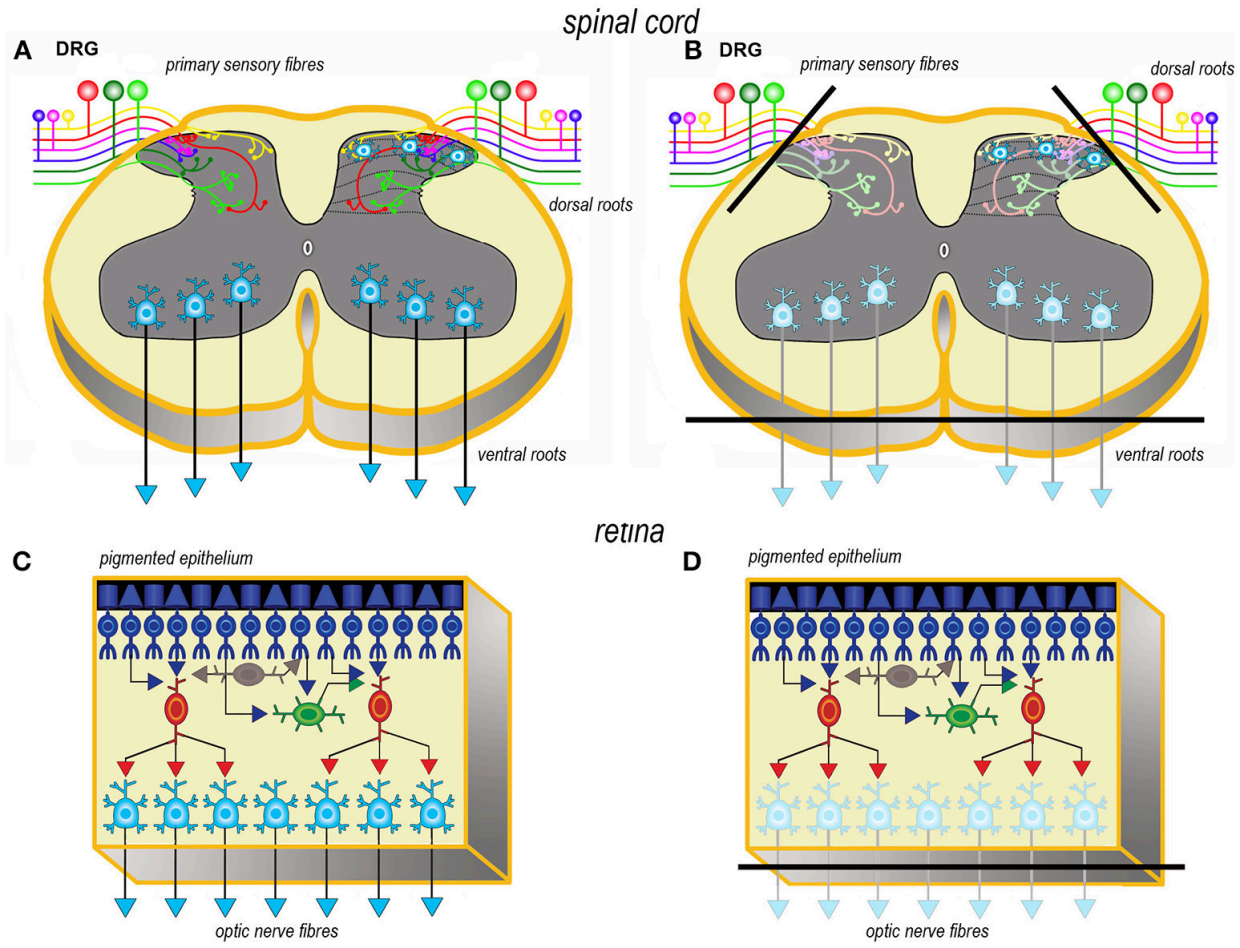
Simplified schematic representations of the effects of the slicing procedure on the preparation of acute slices (left) and OCs (right) from spinal cord **(A, B)** and retina **(C, D)**. Only the main components of circuitry are depicted as well as the principal afferent and efferent connections. In spinal cord **(A, B)** just the interneurons in the superficial dorsal horn and the ventral horn motor neurons are portrayed as the intralaminar and interlaminar circuitries are particularly complex and still not fully understood. The lines of section (solid black bars) of the main fiber systems are represented only in the right panels to show the effects of resection onto the afferent and efferent systems (shadowed in comparison to the corresponding drawings at left). DRG, dorsal root ganglion.

Axotomy of afferent fibers during slicing results in terminal degeneration, transmitter depletion and a profound impact on structure and physiology (Figures 1, 2). The mode of termination of the various afferent systems in different types of slices deeply affects the subsequent structure of OCs (6). In OCs obtained from the cerebral cortex (Figures 1A,B), afferent fibers, mostly originating from the thalamus, primarily reach the granular cells of layers II and IV (25). In hippocampal slices (Figures 1C,D), afferent fibers mainly derive from entorhinal cortex and septum. In cerebellar slices (Figures 1E,F) the afferent mossy and climbing fibers reach, respectively, the granular and molecular layers. In spinal cord (Figures 2A,B), primary afferent fibers derive from the neurons the dorsal root ganglia (DRGs) and terminate in different laminae of the dorsal horn. The retina (Figures 2C,D) represents a remarkable exception, because the entire afferent pathway remains contained within the OC, but the absence of the retinal pigmented epithelium makes less effective the differentiation of the outer segments of the photoreceptors.

##### Axotomy of efferent fibers

Resection of the efferent fibers impacts more or less substantially onto the survival of their parent neurons (6). Efferent fibers from the cerebral cortex (Figures 1A,B) derive from the large pyramidal neurons projecting to the brainstem or the spinal cord, or the cortico-striatal neurons, both residing in layer V. Another important contingent originates from the cortico-thalamic neurons of layer VI. To these, the callosal fibers interconnecting the two hemispheres should also be added. In hippocampus (Figures 1C,D), efferent fibers originate from CA1 and CA3 pyramidal neurons. It is worth noting that the axons of the granule cells (the mossy fibers) undergo a fairly large outgrowth, as a consequence of the slicing procedure (Figure 1D). In cerebellum (Figures 1E,F), efferent fibers originate from neurons in cerebellar nuclei. Efferent projections from cerebellar Purkinje neurons may be refrained from harming if the slice incorporates the nuclear neurons. Remarkably, a few Purkinje neurons deprived of their target develop aberrant self-innervation as a consequence of a massive axonal sprouting. Efferent fibers leaving the spinal cord via the ventral roots of the spinal nerves are cut during the slicing procedure (Figures 2A,B). In the retina (Figures 2C,D), efferent fibers, i.e., the ganglion cells' axons, are all severed in the explant with remarkable consequences on survival.

### Blood vessels and blood-brain barrier (BBB)

Studies carried out after immunocytochemical labeling for some of the proteins of the endothelium have demonstrated that capillaries endure very well culture conditions. Therefore, albeit they are not functional in OCs and there is not blood flow, still endothelial cells produce and secrete several molecules that are capable of influencing the activity of the nerve cells and fibers. This absence of functional capillaries deeply influences the maintenance of OCs in long-term experiments. With time cultures become thinner as a mean to permit a more efficient passive diffusion of oxygen and metabolites. This has outstanding consequences on cytoarchitecture. An important development in perspective is the more and more expanding use of microfluidic chambers to permit an optimal distribution of nutrients and/or to selectively target certain areas of the OC rather than the entire slice (26) and the development of devices for long term perfusion, imaging, and electrical interfacing (27). It is also of relevance the possibility to reconstruct *in vitro* the BBB and use it to condition the medium in which OCs are grown (28).

The BBB can be reconstructed *in vitro* e.g., using co-cultures of bEnd, a mouse immortalized endothelial cell line, and primary astrocytes from the same species in which the two cell populations are maintained separated using Transwell filters as a substrate for cell growth (29).

### Transfection of OCs

Introduction of foreign nucleic acids into cells is commonly referred to as transfection. Initially, the process was developed for DNA, but, nowadays, not only DNA (DNA transfer) but also RNA can be experimentally inserted into cells. As nucleic acids cannot cross the neurolemma, they require to be transported by a carrier (vector) to pass the cellular membrane. As a consequence, a stable or a transient transfection is achieved in relation to the type of nucleic acid introduced into the cell and the type of vector employed in the procedure. The difference between the two is relevant as only in stable transfection the foreign DNA sequence(s) become(s) part host cell genome. Neurons are in general post-mitotic cells. Therefore, as they do not replicate their DNA, it is very difficult to transfect them. For this reason, in most instances gene transfer in OCs results in a transient transfection that, nonetheless, is suitable for experiments lasting from a few days to a few weeks. In general this is not a limitation, as the transgene(s) drive(s) the synthesis of the protein(s) under study for enough time to successfully carry on the majority of *ex vivo* experiments.

#### Biological vectors

##### Viruses

Viruses have a natural capability to cross the cellular membrane during infection. For transfection, viral vectors need to be somewhat modified so that they lose the capability to replicate and cannot destroy infected cells.

The viruses used for gene transfer and stable transfection are Retroviruses, Adeno-associated viruses (AAVs), and Lentiviruses. RNA is the genetic material in Retroviruses. Retroviruses infect only mitotic cells and, therefore, are not suitable for neuronal transfection. The foamy viruses, a subgroup of Retroviruses, are, however, suitable for transfecting the nerve cells (30). Lentiviruses infect both mitotic and non-mitotic cells. They can thus be employed for stable gene transfer in non-dividing mature neurons. To block protein translation it is possible to use a lentivirus that delivers a short hairpin RNA (shRNA) to the transfected cell for obtaining a continual knockdown of gene expression. Using an on-off system with inducible lentiviruses it is possible to dissect out very precisely gene function in targeted neurons (31). The *Herpes simplex* viruses (HSVs) are neurotropic viruses. Their DNA does not integrate into the host genome, but, once introduced into the cell nucleus, it remains there for very long periods and forms an independent fragment of circular DNA that duplicates upon division of the host cell. Therefore, HSVs are particularly useful for long-term experiments.

Adenoviruses possess a double-stranded DNA (dsDNA), whereas in AAVs the genetic material is a single-stranded DNA (ssDNA). These two types of viruses are effective in neuronal transfection as they efficaciously infect both dividing and non-dividing cells. In neurons, Adenoviruses can be employed for transient transfection, whereas the AAVs yield stable transfection.

As viruses usually harbor only a modest quantity of genetic material, some genes are too big to be integrated into the viral genome for transfection. This is obviously a serious drawback of this type of vectors.

##### Plasmids

Plasmids are the most widely-used vectors for DNA transfection of isolated neurons or OCs. Plasmids, also referred to as extra-chromosomal DNAs, consist of a circular dsDNA molecule that remains independent from the host's chromosomal DNA. Their length ranges from a few thousand bp to more than 100 kbp. Plasmids may parasitize or live in symbiosis with yeasts, bacteria, or few cell types from eukaryotes. Before every cell division, both the host-cell chromosomal DNA and the plasmid DNA are duplicated. Then, at cytodieresis, the plasmid DNA is distributed between the two daughter cells, so that the genetic material is propagated along sequential cell generations (32). A great advantage of plasmid vectors is that they can host DNA constructs of small to very big sizes, up to about 20 kbp. However, differently form viruses, plasmids cannot actively penetrate cells and must be delivered by means of *ad hoc* transfection procedures. The naked DNAs make an exception to this general rule. They are particular plasmids capable to cross the cell membrane of at least some types of neurons. Transfection efficiency is low in naked DNAs, but it can be augmented to an high degree by physical means such as ultrasounds and microbubbles (33).

#### Synthetic vectors

Numerous non-biological (synthetic) nanomolecules, such as cationic liposomes, cationic polymers, dendrimers, cyclodextrin, and cell-penetrating peptides (CPPs), can be employed for DNA and RNA delivery to CNS (34). Synthetic vectors are not cytotropic and are more or less cytotoxic. Synthetic vectors are generally used to transfect individual cells after dissociation from tissue explants and/or cell lines, and can be employed *in vivo* after systemic delivery for gene therapy studies (34), or by stereotaxic microinjection in site-specific studies (35, 36). Those most in use are cationic liposomes and cationic polymers.

In water, cationic lipids arrange themselves into spheroidal structures delimited by a double lipid layer and referred to as liposomes (37, 38). When liposomes get in contact with DNA or RNA, they form complexes (lipoplexes) with these molecules. Eukaryotic cells can actively endocytose the lipoplexes, and eventually internalize them within endosomes. Lipoplexes then fuse with the endosomal membrane and are released into the cytosol. Endosomal escape is the capability of a lipoplex to get-away from endosomes. It is one of the most prominent attributes of an efficient synthetic transfection reagent, as the endosomal escape directly correlates with the ability of a given liposome to release its nucleic acid cargo after having been internalized into the host cell. Liposomes can host DNAs or RNAs without limits of molecular size. Cationic liposomes are liposomes made with cationic lipids or obtained after mixing cationic and neutral lipids. They are very cheap to be prepared and display high transfection efficiencies. This explains why cationic liposomes are much diffused in the market.

Polyethylenimine (PEI) and poly-lysine are cationic polymers with a favorable endosomal escape (35, 39, 40). Their structure can be linear or branched, and size strongly affects the transfection efficiency of individual polymers (36, 41, 42).

#### Biolistic transfection

Biolistic transfection (43) relies on a simple physical (mechanical) principle whereby the non-native nucleic acid is introduced into the cells after it is accelerated to acquire enough kinetic energy for crossing their membrane. Usually a plasmid DNA is employed in this type of transfection procedure. The plasmid carries a cDNA sequence of interest and is adsorbed onto the surface of an inert colloidal gold particle that acts as an inactive substance that is a vehicle for the DNA to enter the cell. By this means, the DNA (complexed with gold) reaches a sufficient mass to be propelled by a helium blast and to perforate the cell membrane (44).

Several advantages are associated to the use of biolistics to transfect neurons in OCs. First, only a relatively small amount of DNA, in comparison with other procedures, is necessary for successful transfection. Second, multiple transfections (co-transfection) with different DNAs are easily performed (45, 46). Third, the procedure has high efficiency both *ex vivo* and *in vitro*. Major limitations are the poor penetration of the DNA-gold complex in thick slices and the potential damage to the cell that may eventually die as a consequence of physical overload. Biolistic transfection is very expensive, this being another disadvantage of the procedure.

For animal cell transfection, gold of 1–1.5 μm in diameter (1 μm is, in general, the size of choice) can be used as they do not display obvious differences in efficiency. Comparable labeling efficiency and minimal tissue damage was reported after neuronal transfection with ultra-small gold particles around 40 nm (47).

#### Constructs and reporter molecules

Broadly speaking, transfection methods allow performing two different groups of experiments onto cultured neurons.

One group relies on the classic principles of protein genetic engineering and was the first to be developed. Genetic engineering permits to alter the customary way of operation or behavior of one (single transfection) or more (multiple transfection) proteins inside the cell, this being its most important advantage to the understanding of protein function. Just to give a few examples, it may be possible to overexpress the protein (48), or to reduce/abolish translation by RNA interference (45), or to make neurons synthesizing an alien protein, e.g., a receptor type or subunit that is not normally expressed by that particular type of cell. Altogether, these procedures permit to understand the role of a protein on cell survival, physiology, and pathology. The other group of experiments aims to profit of some distinctive features of the protein artificially introduced into the nerve cell. In the easier use, it is possible to label a given type of neuron by making it to produce a *reporter molecule*, i.e., a protein that can be seen with ease in the microscope or measured by biochemical approaches. To adequately control the reporter's synthesis it is mandatory to employ a general or a cell-specific promoter, according to needs. Reporter molecules are of very different nature, but FRPs are among the more widely diffused.

FRPs exist in different colors: green (green fluorescent protein—GFP), yellow (yellow fluorescent protein—YFP and enhanced YFP - EYFP), red (red fluorescent proteins—RFPs, e.g., DsRed), cyan (cyan fluorescent protein—CFP) and more others. Their numerous variants and mutations are today widely in use as they are amenable to live imagining by several types of fluorescence microscopies (49). FRPs are genetically-encoded fluorescent dyes (GEFDs) that not only label with multiple colors specific populations of neurons, but are of paramount importance in understanding neuronal physiology (50). The most important GEFDs that are in use or have a great potential to be used in OCs experiments can be grouped in five main types, according to their principle of functioning and/or type of information that they permit to achieve. They are briefly described below for a better comprehension of the perspectives for expanding the use *ex vivo* platforms in neuroscience studies that will be discussed in the following section.

*Fluorescence resonance energy transfer (FRET) probes* (consisting e.g., of a pair of FRPs made of the Venus variant of YFP and CFP). The pair can be put together (with FRET) or disjointed (without FRET) by the breaking of a chemical bond in a protein, or as a consequence of a mutual or reciprocal chemical interaction between a ligand and its receptor or two different regions of the same molecule, or after a protein modifies its conformation). FRET is very important as it may be quantified (45, 51, 52).

*Genetically encoded calcium indicators (GECIs)* comprise a number of proteins that sense calcium inside the cell (GCaMP6). Today's GECIs are very sensitive and specific and can detect also very rapid calcium changes (53).

*Optogenetic sensors* can be used to study calcium (Aequorin, Cameleon, GCaMP) or chloride (Clomeleon) fluxes and membrane voltage (54–56).

*Genetically-encoded voltage indicators (GEVIs)* are voltage-sensitive fluorescent proteins (VSFPs) of second (VSFP2s) or third generation (VSFP3s). The first consist of the voltage-sensing domain of a protein isolated by the ascidian *Ciona intestinalis*. The protein is impermeable to calcium, but its voltage sensor is a phosphatase that cuts a FRET pair and thus yields a measurable signal when the intracellular calcium concentration changes as a consequence of a biological event (57). VSFP3s, instead, operate through a FRET-independent mechanism (58, 59).

*Dark-to-bright state fluorescent biosensors* are normally not fluorescent (silent or dark state), but can be rendered fluorescent (bright state) by protein cleavage. Among these biosensors is a type of GFP where fluorescence is fully suppressed by a peptide. As this peptide can be detached from GFP by a protease, the biosensor can be used to study proteolysis in live neurons (60, 61).

## Perspectives for expanding the use *ex vivo* platforms in neuroscience studies

### Reduction of animal number

**Figure 3:**
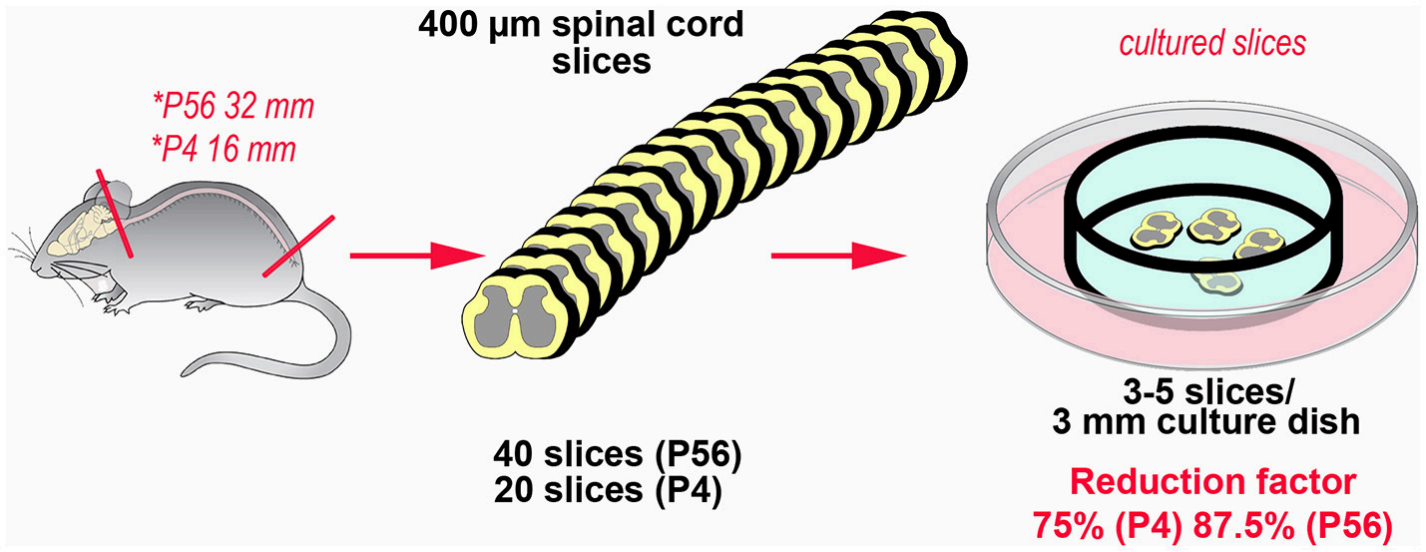
Example of the potential for reduction in the number of experimental animals using slice platforms for *ex vivo* studies. The example is based on the theoretical possibility to use the entire mouse spinal cord after coronal sectioning at a thickness of 400 μm. Rostro-caudal length of the cord is 32 mm at postnatal day 56 and 16 mm at postnatal day 4 (63). In theory it is possible obtaining up to 80 sections/animal (32,000:400) at P56, but a more reliable estimate is around 40 sections/animal. In P4 mice, corresponding figures are 40 (16,000:400) and 20 sections, respectively. It is then possible to cultivate 3–5 slices in 3 cm culture dishes, so that each culture is treated under different experimental conditions. The percentage of reduction thus goes from 75% (P4) to 87.5% (P56).

Abatement of the amount of experimental animals is the main advantage in the use of OCs in neuroscience (62). With substantial differences related to the sources of the slices to be subsequently cultivated *ex vivo*, from one to several tens of fully viable slices can be obtained from a single animal. We have recently estimated that the potential for reduction in our model of spinal cord inflammation may reach 75%, when the P4 spinal cord is put in culture, up to more than 85%, in the case of the adult spinal cord (see Figure 3). Reduction not only is linked to the possibility to cut down the number of subjects euthanized, but also to use slices/OCs obtained from a single animal in several different experiments. In addition, use of slices or OCs raises moderate ethical concerns, because there is no impact on welfare as animals are simply subjected to euthanasia (62).

### Refinement of procedures

Pain and/or distress that are often associated to the most widely used experimental paradigms in neuroscience research are reduced to a minimum (the euthanasic procedure) to prepare slices or OCs, in accordance with the principle of refinement (Table 1). The most relevant applications of OCs under this perspective are discussed below.

**Table 1:** Refinement of procedures in *ex vivo* platforms.

**Type of experiment**	***In vivo***	***Ex vivo***
Cut (deafferentation/deefferentation) and reconstruction of neural connections (nerve regeneration)	Severe (invasive and painful surgery)	Non-recovery
Traumatic lesion	Compression/constriction injury	Non-recovery
Administration of drugs	Moderate (parenteral injection) Severe (intraventricular or intrathecal injection)	Non-recovery
Studies on neurotoxicity	Severe	Non-recovery
Release of biologically active molecules	Severe (microinjection or microelectrode implant)	Non-recovery
Induction of inflammation	Moderate (intradermic injection) Severe (intrathecal injection)	Non-recovery

*Severity is indicated according to current EU regulations (DIRECTIVE 2010/63/EU OF THE EUROPEAN PARLIAMENT AND OF THE COUNCIL of 22 September 2010 on the protection of animals used for scientific purposes)*.

#### Axon regeneration and reconstruction of circuitries

**Figure 4:**
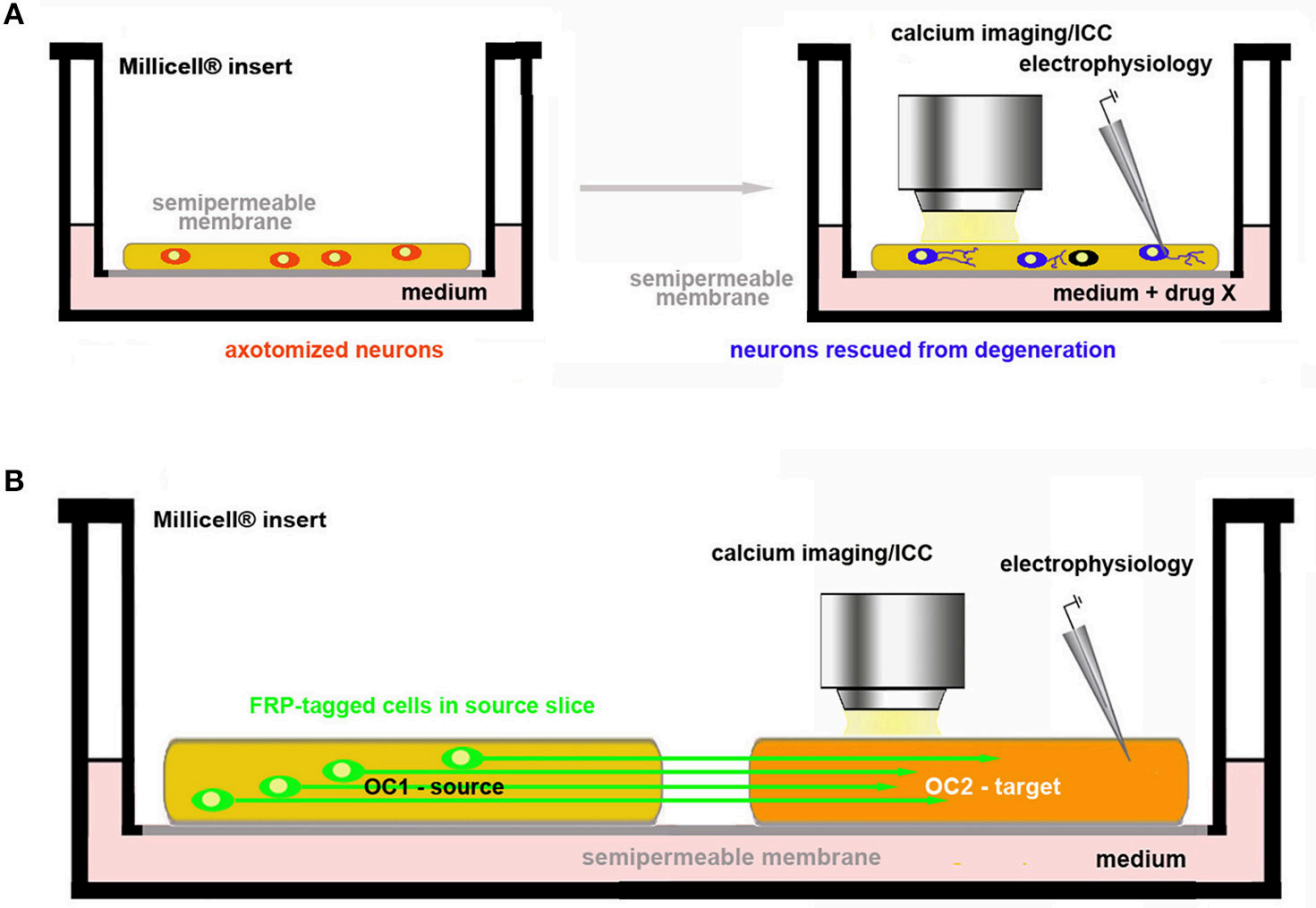
Use of OCs in the study of axon regeneration and reconstruction of circuitries. **(A)** Assessment of drug neuroprotective activity on axotomized neurons in OCs; **(B)** Co-culture of two brain slices. One of the two slices (OC1–source) contains the parent cell bodies of the axons connected with the other slice (OC2–target). These connections are intentionally cut in the preparation of the explant and axonal regeneration is followed in OC2 under control conditions (spontaneous re-innervation) or after pharmacological treatments. In the example, neurons in OC1 are made fluorescent by genetic engineering so that their axons can be easily traced in OC2.

One of the most important areas of investigation in neuroscience research aims to understanding the mechanisms of axon regeneration and to promote the reconstruction of neuronal circuitries lesioned as e.g., consequence of a traumatic event. This is very often performed *in vivo* together with more or less heavy surgical manipulations, severe postoperative consequences, loss of sensorimotor and/or cognitive functions, distress and pain. As mentioned, the slicing procedure necessary to prepare the OCs is always associated with the interruption of afferent and/or efferent connections. This, at first sight, may appear as one of the major limitations to implement the use of *ex vivo* platforms as an alternative to *in vivo*. However, one can take advantage of target disconnection to study neuroprotection and axon regeneration in two main experimental paradigms (Figure 4).

In the first, neurotrophic factors or other putative protective drugs can be added to OCs so that is possible to assess whether these substances may indeed promote neuronal survival and/or axonal growth (Figure 4A). The second option is to use co-cultures of slices obtained from two different regions originally connected to each other and to analyse e.g., the regenerative properties of the molecule(s) of interest (Figure 4B).

##### Protection of axotomized neurons

OCs are valuable tools to study the neuroprotective effects against axotomy-induced cell death (Figure 4A). Several examples can be given to illustrate this type of experiments in slices obtained from hippocampus (64), hypothalamus (65, 66), basal nucleus of Meynert–BNM (23, 67), substantia nigra -SN (68, 69), cerebellum (70–72), retina (73) and DRGs (74). It is interesting to recall here that degeneration of dopaminergic SN neurons and cholinergic BNM neurons is strictly related to development of Parkinson's and Alzheimer's disease, respectively, and that, very recently, a new rat model of Huntington's disease consisting of coronal slices including all the brain regions that may be hit by the pathology has been developed (75). In addition, OCs have been proposed as important tools for medium-throughput screening in neurotoxicological studies (76–78).

##### Reconstruction of axonal connections

To date, OCs are already employed in regeneration studies of neural circuitries. Co-cultures of two (or more) *in vivo* (inter)connected areas of the brain are established to pharmacologically screen the capability of specific molecules to promote axonal growth and rewiring (Figure 4B). References to organotypic co-cultures for studies published before 2009 can be found in Table 1 from Lossi et al. (6). Other more recent reports have been published on the regeneration of specific connections such as those between the sensorimotor cortex and spinal cord (79), the hippocampus and septum (80), the ventral tegmental/area SN and prefrontal cortex/striatum (81, 82). Very interesting, pharmacological screening of molecules promoting axon regeneration was also performed using entorhino-hippocampal (EH) organotypic co-cultures in which the degree of axonal regeneration after EH axotomy was evaluated using entorhinal slices obtained from enhanced green fluorescent protein (EGFP)^+^ transgenics so that regenerating axons were fluorescent (83). A recent study has also developed a two-compartment OC to study nerve recovery after injury (84).

#### Studies on neuroactive/neuroprotective drug candidates and neurotoxic substances

An enormous amount of studies has been and still is devoted to the discovery, synthesis, and characterization of neuroactive substances and/or neuroprotective molecules that have a potential for pharmacological applications. In relation to the principle of Refinement, the benefit of OCs should be considered also under the perspective that most experimental paradigms of this type require induction of experimental neurodegeneration/neuropathology so that live animals and/or transgenic models are first subjected to a heavy treatment in biological and ethical terms (Table 1). Still, current use of OCs raises two important issues. First, slice experiments are often carried out using pharmacological concentrations of the drugs to be tested and offer very limited hints as to the *true* capability of these drugs/molecules to exert an effect, if any, at concentrations that better mimic those to which neurons may be physiologically exposed *in vivo*. Second, they do not permit assessing the capability of the substance(s) under study to cross the BBB.

##### Perspectives for the use of acute slices or OCs in medium-throughput screening

As a future direction, we propose more sophisticated approaches for using OCs in medium-throughput screening studies. Two main lines of application can be envisaged and discussed as follows.

*Pharmacological and physiological screening of neuroactive molecules*. Currently, molecule(s) to be tested *in vitro* is (are) added at different concentrations to culture medium and its (their) effects tested using biochemistry (chromatography, enzyme activity assays), immunochemistry (immunocytochemistry, Western blotting, ELISA), or molecular biology (PCR, RT-PCR, FRET, etc.). With such approaches, it is by no means demonstrable that concentrations giving rise to measurable effects *in vitro* can be effectively reached *in vivo* following physiological cellular release or pharmacological administration.

**Figure 5:**
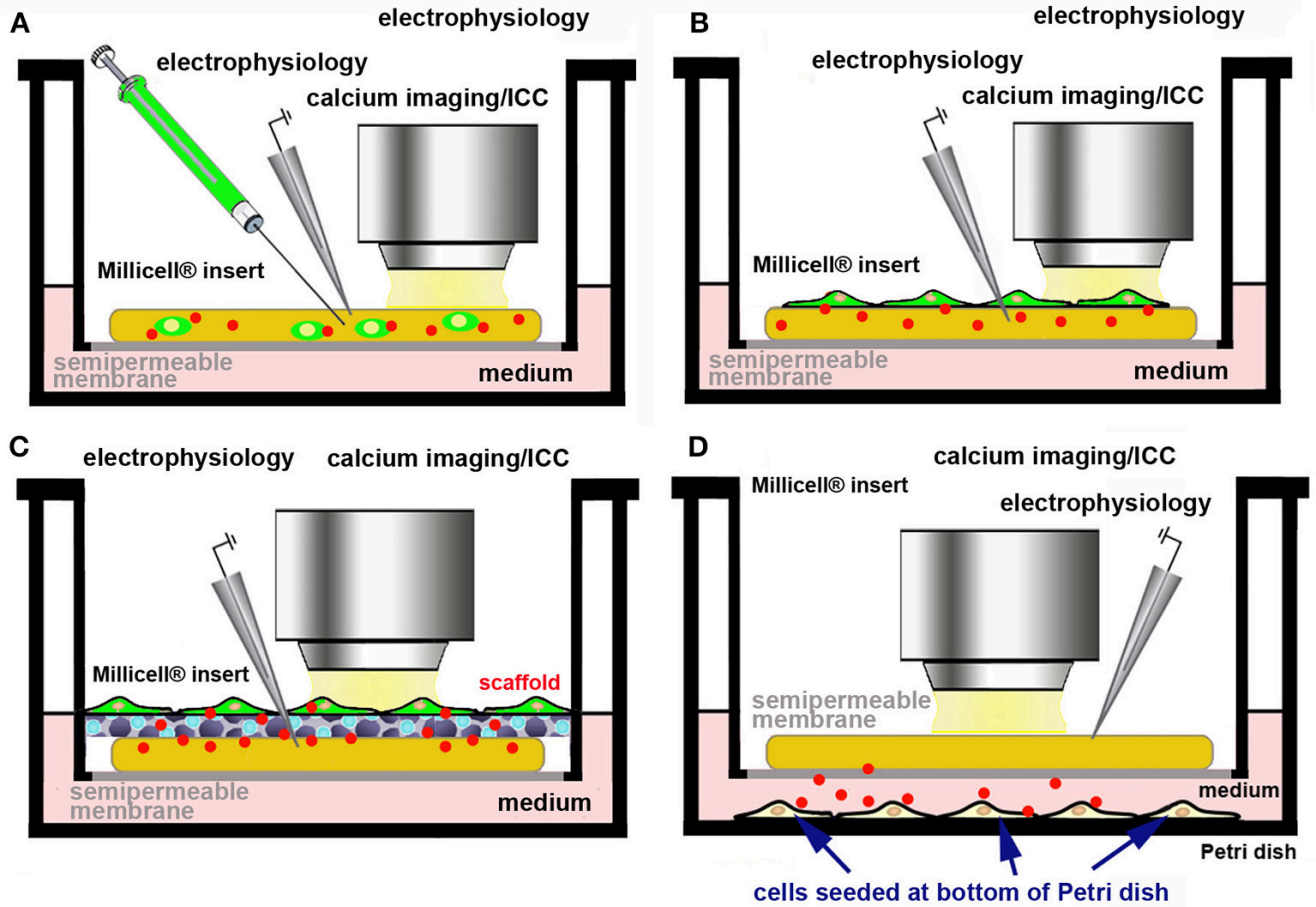
Use of a cell line/primary cells producing a bioactive molecule to assess the effects of its physiological release *ex vivo*. Cells producing the biologically active molecule (red dots) can be made fluorescent by genetic engineering **(A–C)** and directly microinjected into the slice **(A)**, seeded at the top of the OC **(B)**, supported by a scaffold **(C)**, or seeded at the bottom of culture dish **(D)**.

We are currently exploring the use of primary cells and/or cell lines releasing the molecule(s) of interest in the culture medium, physiologically, or after genetic manipulation. Ideally these cells should be made fluorescent to trace their survival and fate within OCs (Figure 5). In practice, fluorescently tagged cells can be directly microinjected (Figure 5A) or seeded (Figure 5B) on the top of OCs. It may also be possible to use an *ad hoc* scaffold to support the foreign cells (Figure 5C) or to seed the cells releasing the molecule of interest at the bottom of the culture dish (Figure 5D). As an example of the use of a scaffold to support and promote the growth of primary cells onto OCs, dopamine-producing human induced-neuronal cells encased in a scaffold were successfully grafted into cortico-striatal OCs, with an amelioration of the growth of processes and increase in the rate of action potentials, when compared to individual cells after dopamine microinjection (85). Scaffolds are also of interest as they offer the possibility to reconstruct *ex vivo* the BBB.

Other approaches already in use for cultures of isolated cells, rely on multiplexing technologies such as Luminex xMAP® multi-analyte profiling, which enables the simultaneous detection, and quantitation of multiple secreted proteins in culture medium and are useful to study the dynamic response of nerve cells to experimentally induced perturbations (86). This high-throughput technology produces results comparable to ELISA but with lower costs, greater efficiency, speed, and dynamic range. Similarly to xMAP®, xTAG® allows easy development/optimization of nucleic acid mid-density arrays that permit evaluating genotypes, gene expression, or miRNAs. However, both approaches have not been used yet in slice or OC applications.

Other interesting opportunities for a more widespread use of OCs are based on the possibility to transfect them with fluorescent probes of various nature (44). Among these, FRET probes permit to study protein-to-protein interactions, proteases (45) or promoters' activity in live cells. With optogenetic probes, instead, individual neurons or neural networks can be activated with flashes of blue light (87). As an example of the potential applications of these probes in pharmacological studies, transplanted dopaminergic neurons differentiated from neural stem cells were demonstrated to functionally integrate in OCs derived from mice with experimentally-induced Parkinson (88).

**Figure 6:**
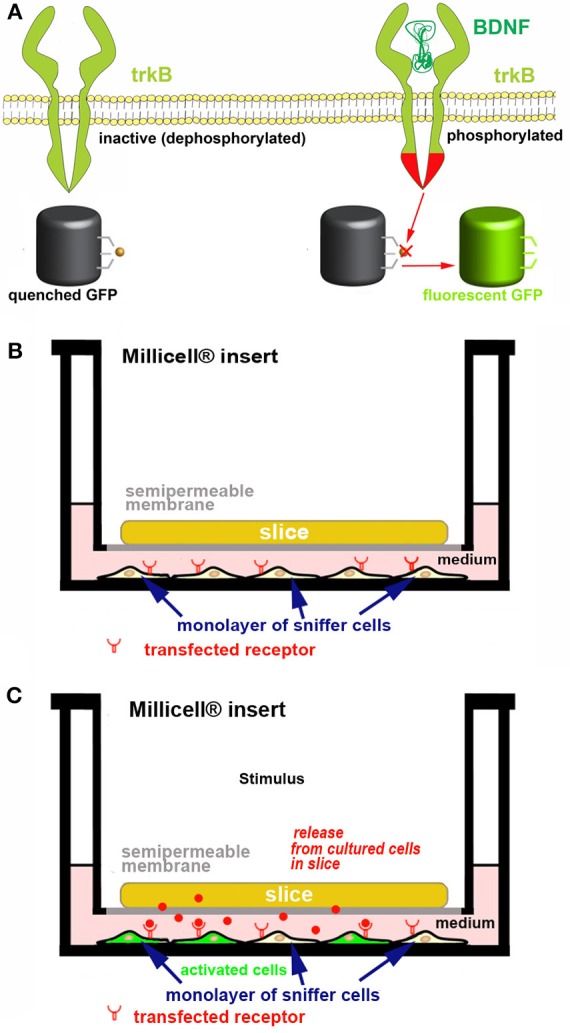
Strategy for developing a new *ex vivo* platform to study the effect of a neurotropic factor. The example describes a co-culture made of an acute or organotypically cultured brain slice and a monolayer of sniffer cells seeded at the bottom of a Petri dish. If an acute slice is used it needs to be kept in place with an *ad hoc* grid (not represented for simplicity). **(A)** engineering of the sniffer cell line to make it suitable for reporting the release of BDNF from slice cells. The sniffer cells are double transfected to express trkB receptors and a dark-to-bright state GFP-based biosensor (60, 61) that is modified to become activated after trkB phosphorylation. **(B**,**C)** Scheme of the co-culture set-up. In **(B)** the sniffer cells are not fluorescent, whereas in **(C)** the BDNF released from the slice (red dots) diffuses through the culture medium, binds to trkB receptors on the sniffer cells and thus some of these cells become fluorescent. The process can be imagined in real time using an inverted wide field or confocal microscope. In the example it is assumed that BDNF release is triggered after an *ad hoc* stimulus.

*Screening endogenous release*. If one is interested in assessing the capability of cultured cells to release one or more bioactive molecules at physiologically relevant concentrations, we propose to use co-cultures made of an OC and a monolayer of responsive “sniffer” cells capable to bind the molecule(s) under study after interaction with specific receptors and to report ligand binding through a fluorescent tag (Figure 6). Activatable GFP, a unique chromophore that remains in a “dark” state because its maturation is inhibited (89) can be used as a reporter molecule. Alternatively, activity based fluorescence biosensors are also interesting (49, 90). Biosensors may e.g. use FRET (91) or consist of polypeptide sequences which undergo a specific physico-chemical modification by the target enzyme, which affects probe fluorescence (90). A very important application lies in non-destructively imaging of tyrosine kinase activities with high spatial and temporal resolution in single living cells to study protein phosphorylation (91). As an example of the potential associated with these probes, we describe in Figure 6 our strategy to monitor *ex vivo* the release and biological activity of brain-derived neurotrophic factor (BDNF).

## Statistical design and data robustness in translational studies using rodent *ex vivo* platforms

One of the main issues in the use of *ex vivo* platforms in neuroscience translational studies regards the soundness and reproducibility of data particularly if one wants to employ these platforms in screening studies as tools for drug development, as there is a strong need to work out instruments that are accepted by regulatory authorities (92). This issue is of more general interest to the point that the European College of Neuropsychopharmacology (ECNP) has promoted, in the recent past, the creation of a Preclinical Data Forum Network (https://www.ecnp.eu/research-innovation/ECNP-networks/List-ECNP-Networks/Preclinical-Data-Forum.aspx).

Among the problems that are recognized to be of primary importance in producing reliable and translationally useful data are those related to poor experimental design and statistical analysis. As reported in a recent publication of the network “There is ample evidence that technical issues are the major drivers. Studies are under-powered, do not follow appropriate blinding and randomization procedures, contain overtly flexible study designs (e.g., insufficiently defined endpoints), use poor statistics and demonstrate an over-reliance on *P*-values (93)”.

If one wants to propose brain slices as alternatives to animal experiments in drug screening, choices in statistical analysis must be strictly performed according to a previously carefully established experimental plan. If not, results cannot be considered at face value. Sample size (i.e., number of animals, number of slices/animal and number of slice/treatment) must be chosen in advance and not adjusted to the results of initial statistics. It was e.g., calculated that there is a 5% chance of error when more data are collected only after a *P* > 0.05 resulted from a first set of experiments (94). Other common mistakes are HARKing (Hypothesizing After the Result is Known) a term introduced by Kerr (95), and the wrong conception that *P*-values give information on effect size, whereas they strictly depend on sample size and must be carefully analyzed in each experimental context (94).

Finally, false positives (type I errors) are very important: if one screens lightly pre-screened molecules expecting that 10% of them indeed have an effect, at 0.80 statistics power and P set at 5% there is a 36% probability to obtain a false positive result (94).

## Concluding remarks

Animal studies still represent a pillar in neuroscience research. The continuous development and amelioration of *ex vivo* approaches will be undoubtedly important in developing new versatile screening tools for translational neuropsychopharmacology and neuroprotective strategies (96, 97). Therefore, in parallel with today's possibility to genetically manipulate slices directly after explant by transfection techniques, OCs also have a potential to further expand in neuroscience research.

## Author contributions

LL wrote the first draft of the manuscript. AM wrote sections of the manuscript. Both authors contributed to manuscript revision, read, and approved the submitted version.

### Conflict of interest statement

The authors declare that the research was conducted in the absence of any commercial or financial relationships that could be construed as a potential conflict of interest. The reviewer IG and handling Editor declared their shared affiliation.
